# *Lilii bulbus* Exerts Anti-Seizure Effects by Modulating GABAergic Synapse Organization in the Pentylenetetrazol Kindling Model

**DOI:** 10.3390/nu18071159

**Published:** 2026-04-04

**Authors:** Hee Ra Park

**Affiliations:** Department of KM Science Research, Korea Institute of Oriental Medicine (KIOM), Daejeon 34054, Republic of Korea; hrpark0109@kiom.re.kr; Tel.: +82-42-868-9519; Fax: +82-42-868-9668

**Keywords:** GABAergic neuron, gephyrin, kindling, *Lilii bulbus*, pentylenetetrazol, seizure

## Abstract

**Background:** We investigated whether a water extract of *Lilii bulbus* (*Lilium lancifolium* Thunberg; WELB) could modulate inhibitory synaptic organization in a mouse model of pentylenetetrazol (PTZ)-induced kindling. **Methods:** Starting 14 days prior to the initial PTZ challenge, WELB (500 mg/kg) was delivered via oral gavage once daily. This treatment regimen was maintained for a total of 40 days, spanning the entire period until the animals reached the fully kindled state. **Results:** Behavioral assessments revealed that WELB treatment significantly reduced seizure severity and Racine scores, prolonged the latency to clonic seizures, and shortened seizure duration, demonstrating potent anticonvulsant activity. Two-photon calcium imaging further showed that WELB markedly suppressed PTZ-induced neuronal hyperexcitability in the posterior parietal cortex, accompanied by decreased expression of neuronal activation markers, including c-fos, phosphorylated-calcium/calmodulin-dependent protein kinase IIα (p-CaMKIIα), and N-methyl-D-aspartate receptor 1 (NR1). In the hippocampus, WELB modulated the expression of GABAergic interneuron markers [glutamate decarboxylase 67 (GAD67), vesicular GABA transporter (VGAT), parvalbumin (PV), somatostatin (SOM)] and upregulated GABAergic gene transcripts [GABA-A receptor α1 subunit (Gabra1), GABA-A receptor α2 subunit (Gabra2), GABA transporter 1 (Gat1), GABA transporter 3 (Gat3), PV, SOM, cholecystokinin (CCK)] that were downregulated by PTZ kindling. Moreover, WELB enhanced the expression of GABAergic synaptic organization-related proteins (gephyrin, collybistin, neurexin-1β, neuroligin-2, and neuropilin-2), indicating its regulatory effect on inhibitory synaptic integrity. **Conclusions:** Collectively, these findings suggest that WELB may exert its anticonvulsant effects by functionally remodeling GABAergic synaptic organization-related factors, thereby restoring inhibitory circuit integrity and providing a mechanism-based therapeutic strategy for epilepsy and seizure-related neurological disorders.

## 1. Introduction

Epilepsy is a chronic neurological disorder characterized by recurrent seizures resulting from an imbalance between excitatory and inhibitory neuronal activity [1]. In general, current antiseizure medications (ASMs) aim to restore this balance primarily by blocking ion channels, modulating neurotransmitter receptors, or reducing glutamatergic neurotransmission. However, these treatments often fail to achieve complete seizure control and frequently associated with adverse effects in patients with epilepsy [2,3,4]. These limitations highlight the urgent need for novel therapeutic strategies that target the molecular mechanisms underlying epileptogenesis, particularly those capable of modulating neuronal hyperexcitability through enhanced inhibitory synaptic innervation.

At the molecular level, the structure and function of GABAergic synapses are tightly regulated by a network of scaffold and adhesion proteins, including gephyrin, neuroligin-2, collybistin, and neurexin-1β [5]. Among these, gephyrin serves as a central postsynaptic scaffold that anchors and stabilizes GABA-A receptors, thereby maintaining inhibitory synaptic efficacy [5]. Neuroligin-2, located on the postsynaptic membrane, interacts with both gephyrin and collybistin to promote gephyrin clustering and proper assembly of inhibitory postsynaptic domains [6]. Collybistin functions as a guanine nucleotide exchange factor that recruits and activates gephyrin at GABAergic synapses [7]. On the presynaptic side, neurexin-1β forms a trans-synaptic complex with neuroligin-2, ensuring precise alignment between presynaptic release sites and postsynaptic receptor clusters [7]. Collectively, these proteins orchestrate the molecular architecture required for stable GABAergic transmission, and their dysfunction has been implicated in impaired inhibitory signaling and increased seizure susceptibility.

Herbal medicines with neuroprotective and pro-neurogenic properties have recently gained attention as potential adjunct or alternative therapies for epilepsy, due to their multi-target mechanisms and relatively low toxicity [8,9]. *Lilii bulbus*, the dried bulbs of *Lilium lancifolium* Thunberg (*Liliaceae*), is a traditional medicinal and functional food widely distributed across East Asia, including Korea, China, and Japan. It has been recognized for its broad pharmacological spectrum, exhibiting potent antioxidant, anti-inflammatory, and anti-cancer properties [10,11,12]. Our previous study demonstrated that the water extract of *Lilii bulbus* (WELB) exerts anticonvulsant effects and alleviates mechanisms associated with epileptogenesis, suggesting its therapeutic potential in epilepsy [13]. However, the precise molecular mechanisms by which WELB modulates inhibitory neurotransmission remain poorly understood.

Building on these findings, we aimed to investigate whether WELB enhances inhibitory synaptic innervation and mitigates neuronal hyperexcitability using a pentylenetetrazole (PTZ)-induced kindling mouse model, a well-established paradigm that recapitulates the progressive development of chronic seizures. Through behavioral assessments, two-photon calcium imaging, Golgi staining, and molecular analyses, we examined whether WELB mitigates seizure severity by modulating GABAergic synaptic organization-related factors.

## 2. Materials and Methods

### 2.1. Preparation of WELB

The raw herbal materials were sourced from Kwangmyungdang Medicinal Herbs Co., (Ulsan, Republic of Korea), having been originally produced in Gansu, China, and imported via Seonglim Pharmaceutical (Bozhou, China). To prepare the water extract of *Lilii bulbus* (WELB), 50 g of the herb was boiled in 1000 mL of distilled water using a heat reflux system for 3 h. Following extraction, the solution was purified through a 25 μm cartridge filter and concentrated via a rotary evaporator under vacuum. The final concentrate was lyophilized into a powder, which was then dissolved in 0.9% physiological saline right before treatment. To ensure consistency and prevent batch-to-batch variation, a single large batch of the WELB extract was prepared, lyophilized, and used for all subsequent experiments in this study.

### 2.2. Animals

Male C57BL/6J mice, aged six weeks, were obtained from Daehan BioLink (Eumseong-gun, Republic of Korea). Prior to any experimental procedures, all animals underwent a 7-day acclimation period under standardized conditions, including a 12 h light/dark cycle (300 lux) and a temperature range of 20–23 °C. The mice were housed in groups of five per cage with unrestricted access to food and water (*ad libitum*). All protocols for animal care were strictly conducted in accordance with the US National Institutes of Health (NIH) guidelines and received formal approval from the Institutional Animal Care Committee of the Korea Institute of Oriental Medicine (Approval No.: #24-027).

### 2.3. Experimental Design and Drug Administration

To induce chronic epilepsy, mice in the PTZ group were administered subconvulsive doses of PTZ (35 mg/kg, i.p.) every 48 h for a 26-day period (totaling 13 injections). Seizure behavior was monitored for 30 min following each administration. Severity was graded according to the modified Racine scale as described by Van Erum et al. [14], which incorporates advanced stages such as wild jumping and tonic extension:

- Stage 0–2: No response (0), behavioral arrest/immobilization (1), or facial jerking (2).
- Stage 3–4: Neck jerks (3) or clonic seizures in a sitting position accompanied by Straub’s tail (4).
- Stage 5–6: Clonic or tonic–clonic seizures while lying on the belly (5) or on the side, including wild jumping (6).
- Stage 7: Tonic extension, which may progress to respiratory failure and mortality.

Mice exhibiting three consecutive seizures at stages 5–6 were considered fully kindled.

A total of 45 mice were randomly assigned to three groups (n = 15 per group):

1. Control (CON)
2. PTZ-kindled group (PTZ; 35 mg/kg, Sigma-Aldrich, St. Louis, MO, USA)
3. PTZ + WELB 500 mg/kg (PTZ + WELB)

**Figure 1:**
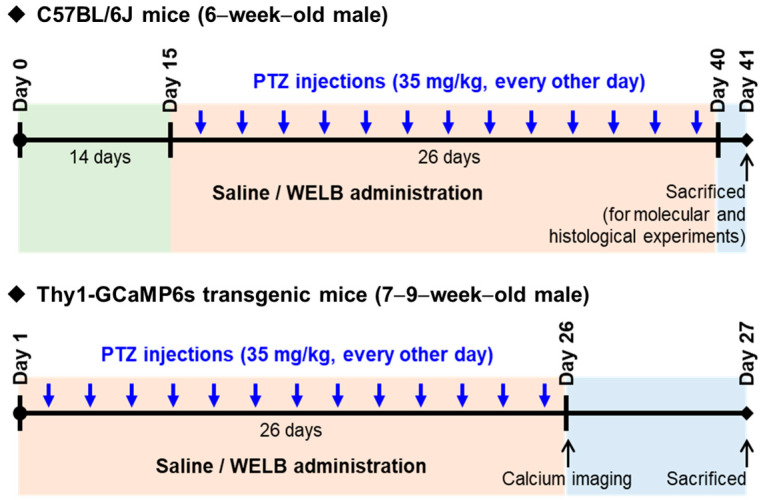
Experimental scheme for the in vivo study. Seven-week-old wild-type C57BL/6J mice (used for behavioral, molecular, and histological analyses) and Thy1-GCaMP6s transgenic mice (n = 3 per group, total n = 9; used for in vivo two-photon calcium imaging) were administered saline (CON and PTZ groups) or WELB (500 mg/kg) orally for a total of 40 days, including 14 days prior to the first PTZ injection and 26 days during the PTZ kindling period until full kindling was achieved. A kindling-induced epilepsy model was established by intraperitoneal injection of PTZ (35 mg/kg) 13 times at two-day intervals. WELB (500 mg/kg) was administered 1 h before each PTZ injection. For C57BL/6J mice, the behavioral responses of the mice were monitored for 30 min following PTZ administration and scored according to the Racine scale, after which the mice were sacrificed for molecular and histological experiments (e.g., Western blotting, RT-qPCR, immunostaining, and Golgi staining). For the Thy1-GCaMP6s mice, mice underwent two-photon calcium imaging to evaluate neuronal hyperexcitability and were sacrificed immediately after the imaging sessions. CON, control; PTZ, pentylenetetrazol; WELB, water extract of *Lilii bulbus*.

All treatments were prepared using 0.9% saline as the vehicle. WELB (500 mg/kg, p.o.) was administered daily to the PTZ + WELB group starting 2 weeks before the first PTZ dose and lasting for 40 days in total. During the seizure induction period, mice received WELB 1 h before each i.p. injection of PTZ. Animals in the CON and PTZ groups were given saline under the same schedule. The experimental protocol is summarized in Figure 1. Group assignment and number of mice per group for in vivo study is presented in Supplementary Materials (S1).

### 2.4. Tissue Preparation

Following the experiment (day 41), mice were euthanized using avertin (2,2,2-tribromoethanol; Sigma-Aldrich). For molecular and biochemical assessments, hippocampal tissues were rapidly isolated and kept at −80 °C. For morphological studies, animals underwent transcardial perfusion with 4% paraformaldehyde in 0.1 M PBS (pH 7.4). Post-perfusion, brains were extracted, fixed overnight in the same solution at 4 °C, and subsequently immersed in 30% sucrose for cryoprotection. Coronal sections (40 μm thickness) were obtained across the rostrocaudal axis (Bregma −2.80 mm to −5.80 mm) using a freezing microtome (Leica Biosystems, Deer Park, IL, USA), guided by the stereotaxic atlas of Allen Mouse Brain Atlas. From the six available series, one set of sections (spaced 240 μm apart) was specifically chosen for each immunostaining procedure. Collected hippocampal sections were preserved at 4 °C in Dulbecco’s PBS supplemented with 0.1% sodium azide.

### 2.5. Golgi Staining

Golgi staining was performed using the FD Rapid GolgiStain™ kit following the manufacturer’s instructions (FD NeuroTechnology, Columbia, MD, USA). Briefly, brains were obtained via cervical dislocation under CO_2_ anesthesia and immersed in impregnation solution for 2 weeks in the dark, followed by immersion in Solution C for 1 week. Cryoprotected brains were coronally sectioned at 100 μm intervals along the rostrocaudal axis and mounted on adhesive-coated slides. Sections were developed using the provided staining solution. Images were acquired with a biological microscope (Olympus, Tokyo, Japan). Spine density was specifically analyzed in the dentate gyrus of the hippocampus. To ensure consistency, only dendrites that were relatively straight, fully impregnated, and distinct from neighboring branches were selected for counting. Dendritic spine density was analyzed in a total of 30 dendrites (six dendrites/mouse; five mice per group).

### 2.6. In Vivo Two-Photon Calcium Imaging of Cortical Neurons in GCaMP6s Transgenic Mice

Thy1-GCaMP6s transgenic mice were used to enable in vivo visualization of neuronal activity. In these mice, the genetically encoded calcium indicator GCaMP6s is expressed under the control of the Thy1 promoter, resulting in predominant expression in excitatory neurons. Changes in intracellular calcium levels were monitored as a proxy for neuronal activity. Calcium imaging was performed in the posterior parietal cortex (PPC), which allows stable and reproducible imaging through a cranial window. The PPC was selected as an accessible cortical region for longitudinal in vivo imaging of neuronal activity. A total of 9 Thy1-GCaMP6s transgenic mice (7–9-week-old male) were randomly assigned to three groups (n = 3 per group: CON, PTZ, and PTZ + WELB). Following the completion of the in vivo imaging sessions, all transgenic mice were immediately sacrificed. Thy1-GCaMP6s transgenic mice were subjected to the PTZ kindling protocol (35 mg/kg, 13 injections, every other day) and administered saline or WELB orally once daily for 2 weeks. Calcium signals in cortical layer II/III neurons of the posterior parietal cortex (PPC) were recorded through cranial windows using a two-photon intravital microscope (IVM-CM3, IVIM Technology Inc., Daejeon, Republic of Korea). Imaging was conducted 1 h after the final PTZ kindling session to visualize neuronal hyperexcitability in PTZ-kindled mice using a 25× objective lens to obtain 35 images from a z-stack of 2 × 2 mosaic fields. The green fluorescence intensity of the calcium signal was quantified from these stacked images. Cluster of differentiation 31–FSD 647 (BioActs, Incheon, Republic of Korea) was injected via the tail vein to label blood vessels.

### 2.7. Immunostaining

To prevent non-specific binding, brain sections underwent a blocking step in Tris-buffered saline (TBS) supplemented with 3% bovine serum albumin (BSA) and 0.1% Triton X-100. The tissues were then incubated with primary antibodies at 4 °C for 24 h. Subsequently, the sections were treated with biotinylated secondary antibodies (1:300; Vector Laboratories Inc., Newark, CA, USA) for a 2 h duration at room temperature. Following a TBS wash, an avidin–biotin complex solution was applied for 1 h. Representative images were captured utilizing an Olympus BX53 optical microscope (Olympus) Primary antibodies used in this study included glutamate decarboxylase 67 (GAD67; 1:1000, Millipore, Waltham, MA, USA), vesicular GABA transporter (VGAT; 1:1000, Thermo Scientific, Waltham, MA, USA), parvalbumin (PV; 1:1000, Synaptic Systems, Göttingen, Germany), and somatostatin (SOM; 1:1000, Abcam, Cambridge, UK). Immunostaining-positive cells for GAD67, VGAT, and PV were counted exclusively in the subgranular zone and granule cell layer of the dentate gyrus, excluding the hilus. Conversely, SOM-positive immunoreactivities were quantified specifically within the hilus of the dentate gyrus. Quantitative analyses of histological data were performed by an investigator (H.R.P.) blinded to the treatment groups.

### 2.8. Western Blot Analysis

Hippocampal tissues were homogenized in RIPA buffer (Millipore) containing a protease inhibitor cocktail (GenDEPOT, Katy, TX, USA) and phosphatase inhibitor cocktail (Roche, Mannheim, Germany), followed by centrifugation at 12,000 rpm for 15 min at 4 °C. The resulting supernatants were stored at −80 °C until analysis. Protein concentrations were determined using a bicinchoninic acid assay kit (Thermo Scientific) with BSA as a standard. Equal amounts of protein (20 μg per lane) were separated by SDS–polyacrylamide gel electrophoresis and transferred to polyvinylidene difluoride membranes (Bio-Rad, Hercules, CA, USA). Membranes were blocked with TBS containing 0.2% Tween-20 and 5% skim milk, incubated with primary antibodies overnight at 4 °C, washed, and then incubated with horseradish peroxidase-conjugated secondary antibodies (1:5000, Thermo Scientific) for 2 h at room temperature. Protein bands were visualized using an enhanced chemiluminescence substrate (SuperSignal™ West Femto, Thermo Scientific) and imaged with a ChemiDoc Touch Imaging System (Bio-Rad). Relative band intensities were quantified using Image Lab software version 6.1.0 (Bio-Rad). Pre-stained protein markers were used to verify molecular weights. Primary antibodies included c-fos (1:1000, rabbit, Santa Cruz Biotechnology, Dallas, TX, USA), phosphorylated Ca^2+^/calmodulin-dependent protein kinase IIα (p-CaMKIIα; 1:1000, Cell Signaling Technology, Danvers, MA, USA), total CaMKIIα (1:1000, Cell Signaling Technology), NMDA receptor 1 (NR1; 1:1000, Invitrogen, Cambridge, MA, USA), metabotropic glutamate receptor 5 (mGluR5; 1:1000, Invitrogen), GAD67 (1:1000, Millipore), gephyrin (1:1000, Synaptic Systems), collybistin (1:1000, Santa Cruz Biotechnology), neurexin-1β (1:1000, Abcam), neuroligin-2 (1:1000, Synaptic Systems), neuropilin-2 (1:1000, Cell Signaling Technology), and α-tubulin (1:5000, Thermo Scientific). Original blots are presented in Supplementary Materials (S2).

### 2.9. Reverse Transcription–Quantitative Polymerase Chain Reaction (RT-qPCR)

Total RNA was extracted from hippocampal tissues using the easy-spin™ Total RNA Extraction Kit (iNtRON Biotechnology, Seongnam-si, Republic of Korea) according to the manufacturer’s protocol. RNA concentration and purity were determined using a NanoDrop One spectrophotometer (Thermo Scientific). Complementary DNA (cDNA) was synthesized from 1 μg of total RNA using the iScript™ cDNA Synthesis Kit (Bio-Rad). Quantitative PCR was performed with iTaq™ Universal SYBR^®^ Green Supermix (Bio-Rad), gene-specific primers, and nuclease-free water on a QuantStudio™ 6 Flex Real-Time PCR System (Life Technologies, Carlsbad, CA, USA). The cycling conditions were as follows: initial denaturation at 95 °C for 30 s, followed by 40 cycles of denaturation at 95 °C for 15 s and annealing/extension at 60 °C for 1 min. GAPDH served as the internal control for normalization. The relative mRNA expression levels were calculated using the standard 2^−ΔΔ*CT*^ method. The expression of each target gene was normalized to the endogenous control (GAPDH), and the relative fold changes were compared to the CON group. All primers were synthesized by Bioneer Corporation (Daejeon, Republic of Korea), and their sequences are listed in Table 1.

### 2.10. Statistical Analyses

Data were evaluated using an unpaired Student’s *t*-test and two- or one-way analysis of variance followed by an appropriate Tukey’s test. Analyses were performed using GraphPad PRISM software^®^ (GraphPad PRISM software Inc., Version 9.5.1, San Diego, CA, USA). The results are expressed as means ± standard errors of the mean. Statistical significance was set at *p* < 0.05. As a single-investigator study, behavior observations were performed unblinded. However, to eliminate observer bias, other experiments were strictly blinded. An independent colleague coded the samples with randomized numbers, and unblinding occurred only after all data analyses were completed. The minimal dataset and the full matrix of *p*- and F-values for all pairwise comparisons are presented in Supplementary Materials (S3).

## 3. Results

### 3.1. WELB Exerts Protective Effects Against PTZ-Induced Seizure Kindling

**Figure 2:**
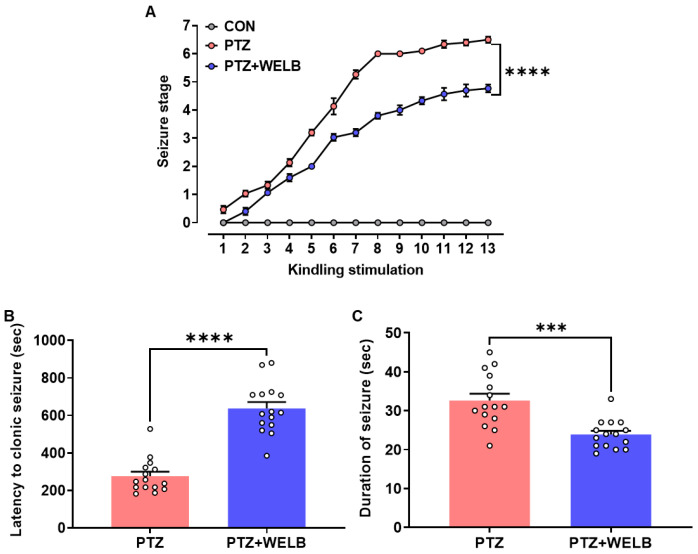
Anticonvulsant effect of WELB in PTZ kindling-induced seizures. (**A**) Racine score assessment showing the effect of WELB on seizure progression during PTZ kindling. Data are expressed as means ± SEMs (n = 15 mice/group). Statistical significance was determined using two-way ANOVA followed by Tukey’s test. (**B**) Latency to clonic seizure in mice after the 13th PTZ injection. (**C**) Duration of clonic seizure after the 13th PTZ injection. Data are expressed as means ± SEMs (n = 15 mice/group). Statistical significance was determined using unpaired Student’s *t*-test. *** *p* < 0.001, **** *p* < 0.0001. CON, control group; PTZ, pentylenetetrazol; SEM, standard error of the mean; WELB, water extract of *Lilii bulbus*.

To investigate the potential of WELB in mitigating seizure activity, seizure intensity was monitored for 30 min following each PTZ challenge using the Racine scale. The group receiving WELB treatment showed significantly attenuated Racine scores throughout the kindling period compared to the PTZ-only group (Figure 2A). At the 13th PTZ stimulation, the latency to clonic seizures and duration of seizure episodes were analyzed. PTZ-kindled mice showed a latency to clonic seizure of 276.07 ± 23.60 s following PTZ injection, whereas WELB-treated mice exhibited a significantly prolonged latency of 637.13 ± 34.43 s (Figure 2B). Moreover, the seizure duration was reduced from 32.60 ± 1.76 s in the PTZ group to 23.87 ± 0.95 s in the WELB-treated group (Figure 2C). Collectively, these results indicate that WELB treatment significantly prolongs seizure latency and shortens seizure duration, demonstrating a potent anticonvulsant effect against PTZ-induced kindling.

### 3.2. WELB Treatment Suppresses PTZ Kindling-Induced Neuronal Hyperexcitability in the Brain

**Figure 3:**
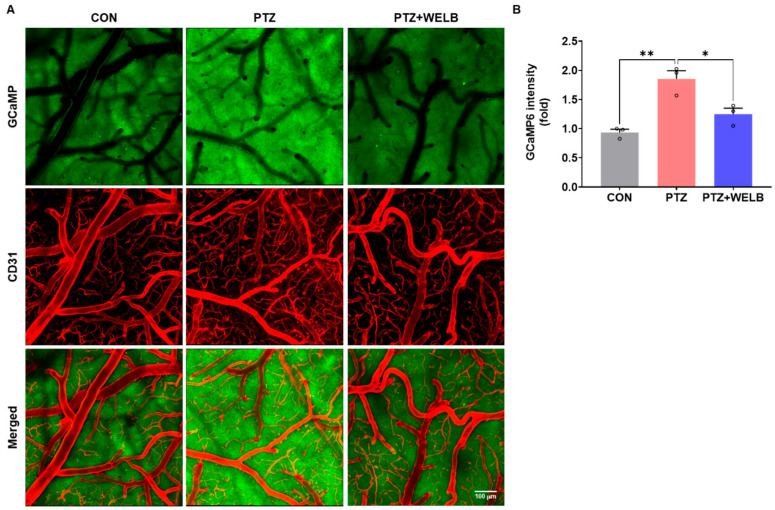

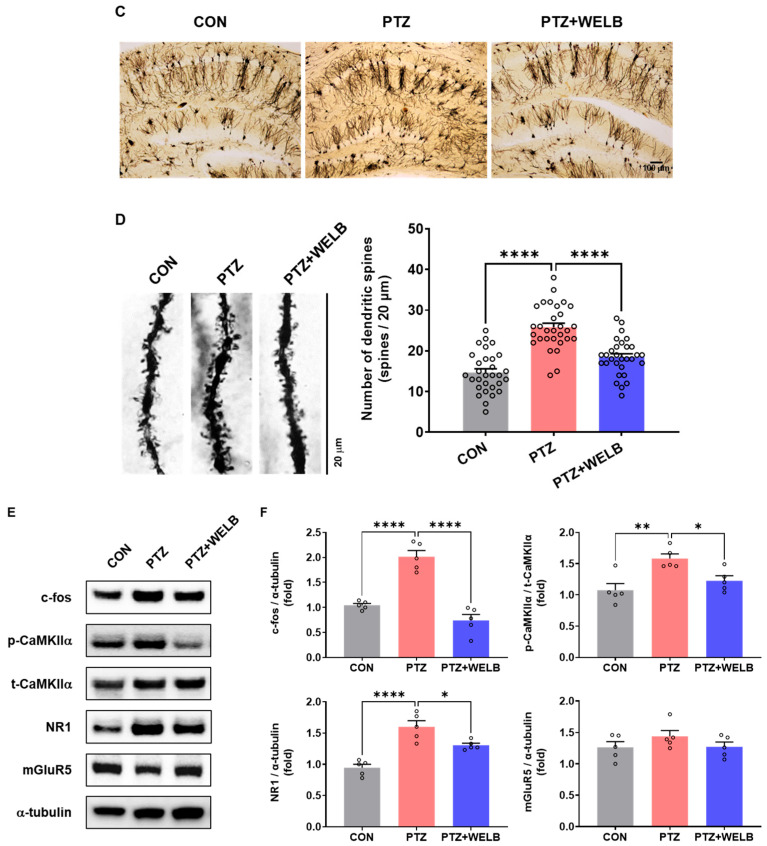
Effect of WELB on PTZ kindling-induced neuronal hyperexcitability in the brain. (**A**) Representative in vivo two-photon calcium imaging in the posterior parietal cortex (PPC) of GCaMP6s transgenic mice. Green indicates GCaMP signal; red indicates CD31 (a marker for blood vessels). Scale bar = 100 μm. (**B**) Quantification of GCaMP fluorescence intensity in each group. Data are expressed as mean ± SEM (n = 3 mice/group); * *p* < 0.05, ** *p* < 0.01. (**C**) Representative images of Golgi staining in the DG. Scale bar = 100 μm. (**D**) Representative Golgi staining images in the dentate gyrus. Scale bar = 20 μm. Quantification of the number of dendritic spines in each group. Data are presented as means ± SEMs (n = 30 dendritic spines from 5 mice). **** *p* < 0.0001. (**E**) Representative Western blots of c-fos, p-CaMKIIα, t-CaMKIIα, NR1, mGluR5, and α-tubulin in the hippocampus. (**F**) Densitometric quantification of protein expression ratios (c-fos/α-tubulin, p-CaMKIIα/t-CaMKIIα, NR1/α-tubulin, and mGluR5/α-tubulin). Data are expressed as means ± SEMs (n = 5 mice/group). Statistical significance was determined using one-way ANOVA followed by Tukey’s test.; * *p* < 0.05, ** *p* < 0.01, **** *p* < 0.0001. CaMKIIα, calcium/calmodulin-dependent protein kinase IIα; CD31, cluster of differentiation 31; CON, control group; GCaMP, genetically encoded calcium indicator; mGluR5, metabotropic glutamate receptor 5; NR1, N-methyl-D-aspartate receptor subunit 1; p-, phosphorylated-; PTZ, pentylenetetrazol; SEM, standard error of the mean; t-, total-; WELB, water extract of *Lilii bulbus*.

To investigate whether WELB modulates neuronal hyperexcitability during PTZ-induced seizures, we examined calcium dynamics in the PPC of Thy1-GCaMP6s mice using two-photon calcium imaging. The PPC is functionally connected with the hippocampus in both anatomical and network terms [15]. Therefore, we investigated the efficacy of WELB on neuronal hyperactivity in PTZ-kindled mice by performing calcium imaging in the PPC and Golgi staining in the hippocampus. Analysis revealed a significant increase in green fluorescence intensity in PTZ-kindled mice compared with CON mice (Figure 3A,B). Notably, WELB treatment markedly attenuated the PTZ-induced elevation in calcium signals (Figure 3A,B). To further evaluate the effects of WELB on neuronal morphology, Golgi staining was performed. PTZ-kindled mice exhibited a greater number of dendritic spines in the dentate gyrus compared with CON mice (Figure 3C,D). WELB treatment significantly reduced the number of dendritic spines that were excessively increased by PTZ kindling. Next, to determine whether WELB mitigates neuronal excitation in the hippocampus, we assessed the expression of neuronal activation markers, including c-fos, CaMKIIα, NR1, and mGluR5. Compared with the CON group, PTZ-kindled mice showed significantly elevated expression of c-fos, p-CaMKIIα, and NR1 (Figure 3E,F). However, WELB treatment markedly reduced these protein levels in the hippocampus (Figure 3E,F), indicating that WELB effectively suppresses PTZ kindling-induced neuronal hyperactivity in the hippocampus.

### 3.3. WELB Is Associated with Preservation of GABAergic Interneuron Expression in the Hippocampus of PTZ-Kindled Mice

**Figure 4:**
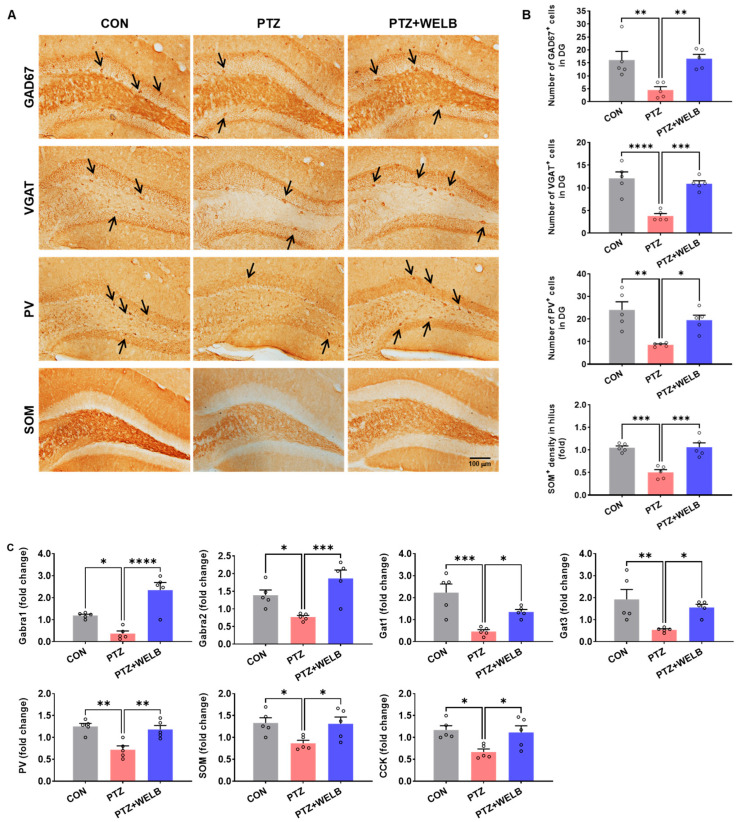
Effects of WELB on the expression of GABAergic interneurons in the hippocampus of PTZ kindling-induced seizure mice. (**A**) Representative immunofluorescence images showing GAD67, VGAT, PV, and SOM expression in the hippocampus. Arrows indicate representative immunostaining-positive cells that were included in the quantitative analysis. Scale bar = 100 μm. (**B**) Quantification of the number or fluorescence intensity of immunoreactive cells in each group. (**C**) Quantification of mRNA expression levels of GABAergic interneuron-related genes (Gabra1, Gabra2, Gat1, Gat3, PV, SOM, and CCK) in the hippocampus. Data are expressed as means ± SEMs (n = 5 mice/group). Statistical significance was determined using one-way ANOVA followed by Tukey’s test.; * *p* < 0.05, ** *p* < 0.01, *** *p* < 0.001, **** *p* < 0.0001. CCK, cholecystokinin; CON, control group; DG, dentate gyrus; Gabra1, gamma-aminobutyric acid (GABA)-_A_ receptor alpha 1 subunit; Gabra2, GABA-_A_ receptor alpha 2 subunit; GAD67, glutamate decarboxylase 67; Gat1, GABA transporter 1; Gat3, GABA transporter 3; PTZ, pentylenetetrazol; PV, parvalbumin; SEM, standard error of the mean; SOM, somatostatin; VGAT, vesicular GABA transporter; WELB, water extract of *Lilii bulbus*.

To elucidate the molecular mechanisms underlying the anticonvulsant effects of WELB, we examined its effect on GABAergic interneurons in the hippocampus of PTZ-kindled mice. The PTZ group showed a marked reduction in the expression of inhibitory neuronal markers, including, GAD67, VGAT, PV, and SOM (Figure 4A,B). WELB treatment increased the expression of these markers (Figure 4A,B). Consistent with these findings, mRNA expression analysis revealed that PTZ-kindled mice exhibited significantly reduced levels of GABAergic interneuron-related genes, including Gabra1, Gabra2, Gat1, Gat3, PV, SOM, and CCK in the hippocampus (Figure 4C). WELB treatment significantly increased the expression of these genes (Figure 4C), further supporting its restorative effect on GABAergic neurotransmission.

### 3.4. WELB Modulates GABAergic Synaptic Regulatory Mechanisms in the Hippocampus of PTZ-Kindled Mice

**Figure 5:**
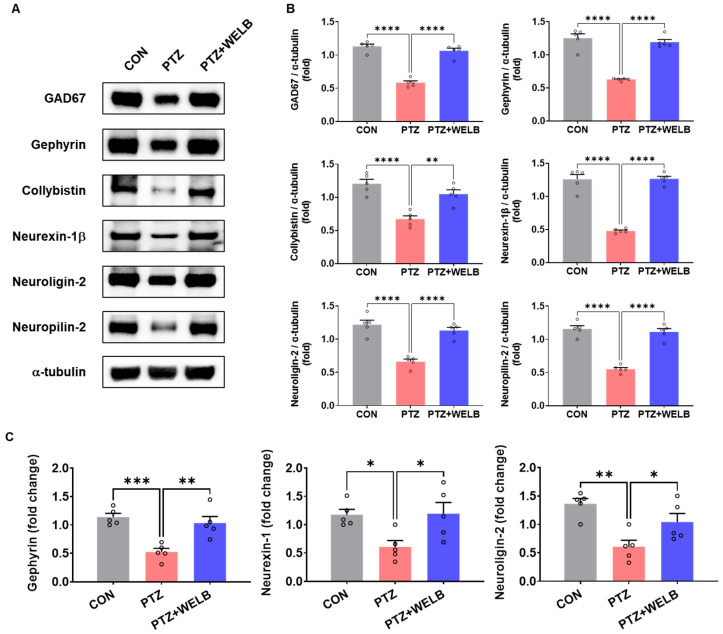
Effects of WELB on the expression of GABAergic synaptic organization-associated factors in the hippocampus of PTZ kindling-induced seizure mice. (**A**) Representative Western blots of GAD67, gephyrin, collybistin, neurexin-1β, neuroligin-2, neuropilin-2, and α-tubulin in the hippocampus. (**B**) Densitometric quantification of protein expression ratios (GAD67/α-tubulin, gephyrin/α-tubulin, collybistin/α-tubulin, neurexin-1β/α-tubulin, neuroligin-2/α-tubulin, and neuropilin-2/α-tubulin). (**C**) Quantification of mRNA expression levels of gephyrin, neurexin-1, and neuroligin-2 in the hippocampus. Data are expressed as the mean ± SEM (n = 5 mice/group). Statistical significance was determined using one-way ANOVA followed by Tukey’s test.; * *p* < 0.05, ** *p* < 0.01, *** *p* < 0.001, **** *p* < 0.0001. CON, control group; GAD67, glutamate decarboxylase 67; PTZ, pentylenetetrazol; SEM, standard error of the mean; WELB, water extract of *Lilii bulbus*.

To further explore the molecular mechanisms underlying the anticonvulsant and neuroprotective effects of WELB in PTZ-induced kindling, we analyzed the expression of GABAergic synaptic organization-related proteins in the hippocampus. As shown in Figure 5A,B, PTZ kindling significantly decreased the expression of GAD67, gephyrin, collybistin, neurexin-1β, neuroligin-2, and neuropilin-2 compared with the CON group. WELB treatment effectively increased the expression of these synaptic proteins (Figure 5A,B). Similarly, mRNA expression of *gephyrin*, *neurexin-1*, and *neuroligin-2* was markedly reduced in the PTZ group compared with the CON group but significantly upregulated by WELB treatment in the hippocampus (Figure 5C). These findings suggest that WELB exerts its anticonvulsant effects, at least in part, by preserving GABAergic synaptic assembly and stabilization in the hippocampus.

## 4. Discussion

In this study, we demonstrated that WELB exerts potent anticonvulsant effects in a PTZ-induced kindling model of epilepsy by attenuating neuronal hyperexcitability and restoring GABAergic synaptic organization-related factors. These findings extend our previous report on the anticonvulsant activity of WELB by demonstrating its association with the preservation of molecular markers at inhibitory GABAergic synapses. While the potential effects of WELB on specific ion channels remain to be determined, the present data demonstrate that its administration attenuates the PTZ-induced loss of key proteins associated with GABAergic synaptic organization. Although numerous AEDs have been developed, their effects are mainly symptomatic. Our data suggest that WELB targets the underlying synaptic pathology, addressing one of the critical unmet needs in epilepsy treatment.

Our previous phytochemical analysis identified p-coumaric acid and ferulic acid as major phenolic constituents of WELB [13]. These compounds are known to exert antioxidant and neuroprotective actions and have been reported to modulate neuronal excitability. Notably, both phenolic acids have been associated with enhancement of GABAergic signaling, either by attenuating glutamate-driven excitotoxicity or by supporting GABA-A receptor function [16,17]. Their presence in WELB provides a plausible mechanistic basis for the observed restoration of GABAergic interneuron markers and synaptic scaffold proteins in PTZ-kindled mice. Although WELB is a multi-component extract, these bioactive phenolics may contribute synergistically to its overall anticonvulsant activity.

Epilepsy is a chronic neurological disorder characterized by recurrent and unpredictable seizures resulting from aberrant, synchronized neuronal discharges in the brain. The maintenance of brain excitability relies on a delicate balance between excitatory glutamatergic and inhibitory γ-aminobutyric acid (GABA)ergic signaling. Disruption of this balance, particularly through the loss of GABAergic inhibition, is a fundamental hallmark of seizure generation and propagation [18]. Although numerous AEDs have been developed over the past decades, most primarily act by modulating ion channels or neurotransmitter release, providing only symptomatic control rather than addressing the underlying pathophysiology [19,20,21,22]. Consequently, approximately one-third of patients remain refractory to current therapies, and long-term treatment is frequently limited by adverse effects and drug tolerance [23,24]. These limitations highlight the urgent need for novel therapeutic strategies that precisely target the molecular mechanisms underlying epileptogenesis. A deeper understanding of the cellular and synaptic processes governing neuronal hyperexcitability-particularly those involving the imbalance between excitatory and inhibitory neurotransmission-will be essential for developing more effective mechanism-based interventions for epilepsy.

PTZ kindling significantly increased neuronal hyperexcitability in both the PPC and hippocampus, as evidenced by calcium imaging and Golgi staining. Epileptiform activity induced by PTZ kindling is not restricted to a single brain region but propagates across distributed cortical–hippocampal circuits, reflecting the network-level nature of epilepsy. Although calcium imaging was performed in the PPC while molecular analyses were focused on the hippocampus, these regions are functionally interconnected and contribute to large-scale brain network dynamics. Therefore, calcium imaging in the PPC provides a meaningful readout of neuronal hyperexcitability at the network level, whereas hippocampal analyses offer mechanistic insights into synaptic alterations. Previous studies support strong functional coupling between the hippocampus and parietal cortex. Human electrophysiological recordings demonstrate bidirectional directed influence between hippocampus and parietal cortex during memory encoding and recall, indicating integrated activity across these regions [15]. Functional MRI studies also support coupling between hippocampal and parietal regions in cognitive tasks [25] and show that the hippocampus participates in large-scale networks incorporating posterior parietal/precuneus nodes such as the default mode network [26]. These findings indicate that cortical imaging of PPC can provide meaningful insight into broader hippocampo-cortical network dysfunction in PTZ kindling.

PTZ kindling induced a marked downregulation of essential inhibitory neuronal markers (GAD67, VGAT, PV, SOM) and GABAergic genes (*Gabra1*, *Gabra2*, *Gat1*, *Gat3*, *CCK*). This disruption of components critical for maintaining inhibitory tone is highly consistent with previous findings [27,28,29,30]. Furthermore, the reduction in the core scaffolding protein gephyrin aligns with established reports of disrupted GABAergic transmission following seizure-induced excitotoxicity [31]. Notably, recent evidence has underscored the critical importance of specific synaptic proteins in this context, demonstrating that the preservation or overexpression of collybistin strongly protects against PTZ-induced seizures and enhances GABAergic neurotransmission [32]. Building upon these foundational studies, the present data demonstrate the concurrent downregulation of the extended structural complex—specifically involving collybistin and neurexin-1β alongside gephyrin and neuroligin-2. The coordinated disruption of this entire gephyrin–neuroligin–collybistin–neurexin axis in the chronic PTZ kindling model has not been extensively characterized. Thus, these findings not only corroborate the known vulnerability of GABAergic networks to PTZ-induced damage but also provide a novel, comprehensive view of the profound synaptic structural degradation occurring in this model.

Loss-of-function studies of GAD67, VGAT, PV, and SOM have consistently demonstrated increased seizure susceptibility, underscoring their critical roles in seizure control. Several studies have shown that alterations in these genes lead to epileptic phenotypes. GAD67 is crucial for maintaining basal GABA levels in the brain [33]. Knockdown of GAD67 in the amygdala disrupts fear extinction- and anxiety-related behaviors, and induces spontaneous seizures, indicating its essential role in inhibitory neurotransmission [34]. VGAT is responsible for packaging GABA into synaptic vesicles [35]. Dusing et al. reported that deletion of VGAT-expressing interneurons in the hippocampus resulted in dramatic seizure clusters and persistent epileptiform activity [36]. PV- and SOM-expressing interneurons are key regulators of network excitability [37], and loss of these interneurons has been associated with increased seizure susceptibility [37,38]. Mutations in *Gabra1* and *Gabra2*, which encode subunits of the GABA-A receptor, disrupt GABAergic circuits by impairing the clustering of gephyrin and neuroligin-2 [39]. *Gat1* and *Gat3*, which encode GABA transporters, are important for regulating extracellular GABA levels and preventing seizure activity [40,41]. Furthermore, recurrent seizures often cause a reduction in CCK-positive inhibitory interneurons in the hippocampus, leading to decreased inhibition of neuronal activity and heightened seizure susceptibility [42,43]. Therefore, the robust restoration of these critical inhibitory neuronal markers and GABAergic genes by WELB indicates a profound recovery of interneuron function. This reestablishment of the excitatory-inhibitory balance within hippocampal circuits strongly supports the therapeutic potential of WELB in mitigating seizure susceptibility.

The observation that WELB treatment increases the expression of key GABAergic synaptic proteins—gephyrin, collybistin, neuroligin-2, and neurexin-1β suggests that WELB may counteract the synaptic deficits induced by PTZ kindling. These proteins are essential for the formation and maintenance of inhibitory synapses, and their dysfunction has been implicated in various forms of epilepsy. Gephyrin serves as a core postsynaptic scaffolding protein essential for anchoring and clustering GABA-_A_ receptors at inhibitory synapses, thereby maintaining efficient inhibitory neurotransmission. Genetic deletion or mutation of gephyrin disrupts receptor clustering and induces neuronal hyperexcitability and seizure susceptibility in mice [44,45]. Collybistin, a guanine nucleotide exchange factor, recruits and stabilizes gephyrin at inhibitory synapses, facilitating the assembly of functional GABAergic postsynaptic structures. Mice lacking collybistin exhibit marked reductions in gephyrin and GABA-_A_ receptor clusters, leading to impaired synaptic inhibition and seizure-like phenotypes [46]. Neuroligin-2, a postsynaptic adhesion molecule, forms trans-synaptic complexes with presynaptic neurexins to regulate the formation and function of inhibitory synapses. Neuroligin-2 knockout mice display diminished inhibitory postsynaptic currents and heightened seizure susceptibility, underscoring its critical role in maintaining the excitation-inhibition balance [47]. Neurexin-1β, a presynaptic adhesion protein, interacts with neuroligin-2 to align presynaptic release machinery with postsynaptic receptor sites, ensuring proper synaptic communication. Disruption of neurexin-1β or its interaction with neuroligin-2 impairs GABAergic synaptic transmission and has been associated with increased seizure activity and epileptiform discharges [48]. Mechanistically, our findings indicate that WELB acts on the gephyrin–neuroligin–collybistin–neurexin axis, which orchestrates the clustering of GABA-_A_ receptors and stabilizes inhibitory synapses. By normalizing the expression of these synaptic proteins, WELB may modulate inhibitory homeostasis, reduce network hyperexcitability, and prevent the progression of epileptogenesis. Collectively, these results suggest that WELB stabilizes the gephyrin–neuroligin–collybistin–neurexin complex, a molecular scaffold essential for inhibitory GABAergic synaptic assembly, thereby mitigating network hyperexcitability.

Despite these promising findings, several limitations warrant consideration. First, in the present study, the kindling acquisition phase was considered complete at the 13th injection, as the PTZ group met the predefined criterion for full kindling (three consecutive seizures at stages 5–6). However, because the protocol concluded at this point without extended injections (e.g., 20–25 injections, which caused severe mortality in our preliminary observations) or a subsequent washout challenge test, it remains unclear whether WELB exerts a true antiepileptogenic effect or merely delays the kindling process (an anticonvulsant effect). Furthermore, while our biochemical data clearly demonstrate the preservation of GABAergic synaptic proteins, these findings are fundamentally descriptive. The apparent restoration of these proteins in the WELB group cannot be definitively attributed to a direct mechanistic action of the WELB. An alternative and highly plausible interpretation is that the lack of protein degradation is secondary to the reduced seizure severity experienced by the WELB treatment by the 13th injection. Future studies employing matched-seizure severity comparisons will be necessary to definitively separate the direct molecular effects of WELB from secondary neuroprotective outcomes. The specific bioactive compounds in WELB responsible for these effects remain unidentified, and their pharmacokinetic and pharmacodynamic properties require further characterization. Moreover, while the PTZ kindling model recapitulates key features of chronic seizures, additional studies using other models, including genetic or spontaneous epilepsy models, are needed to generalize these findings. Finally, long-term safety and efficacy studies are necessary to evaluate the translational potential of WELB for clinical applications. Future investigations employing electrophysiological recordings or conditional knockdown models will be essential to determine whether WELB directly modulates GABAergic synaptic plasticity.

## 5. Conclusions

In conclusion, our study demonstrates that WELB exerts significant anticonvulsant effects in PTZ-kindled mice by suppressing neuronal hyperexcitability and restoring GABAergic synaptic integrity. These findings highlight the potential of WELB as a multifaceted neuroprotective agent and support its further development as a novel therapeutic candidate for epilepsy and related neurological disorders.

## Figures and Tables

**Table 1:** Primer sequences used for RT-qPCR.

Name	Primer and Sequence (5′-3′)
*Gabra1*	Forward-CTCTCCCACACTTTTCTCCC Reverse-CCGACAGTGTGCTCAGAATG
*Gabra2*	Forward-AGATTCAAAGCCACTGGAGG Reverse-CCAGCACCAACCTGACTG
*Gat1*	Forward-TAACAACAACAGCCCATCCA Reverse-GGAGTAACCCTGCTCCATGA
*Gat3*	Forward-CTATGATGCCCCTCTCTCCAC Reverse-CTGTCACAAGACTCTCCACG
*PV*	Forward-TGCTCATCCAAGTTGCAGG Reverse-GCCACTTTTGTCTTTGTCCAG
*SOM*	Forward-AGGACGAGATGAGGCTGG Reverse-CAGGAGTTAAGGAAGAGATATGGG
*CCK*	Forward-ATACATCCAGCAGGTCCGCAA Reverse-CAGACATTAGAGGCGAGGGGT
*Gephyrin*	Forward-GACAGAGCAGTACGTGGAACTTCA Reverse-GTCACCATCATAGCCGTCCAA
*Neurexin-1*	Forward-AACGGACTGATGCTTCACACA Reverse-GATATTGTCACCTGACGCAGATT
*Neuroligin-2*	Forward-TTCCCACCACTCAGAAGGAC Reverse-GTGCTGTCTTCTCGGTCACA
*GAPDH*	Forward-CCTCGTCCCGTAGACAAA Reverse-AATGAAGGGGTCGTTGATG

CCK, cholecystokinin; Gabra1, gamma-aminobutyric acid (GABA) receptor alpha 1; Gabra2, GABA receptor alpha 2; Gat1, GABA transporter type 1; Gat3, GABA transporter type 3; GAPDH, glyceraldehyde-3-phosphate dehydrogenase; PV, parvalbumin; SOM, somatostatin.

## Data Availability

The original contributions presented in this study are included in the article/Supplementary Material. Further inquiries can be directed to the corresponding author.

## Supplementary Materials

The following supporting information can be downloaded at: https://www.mdpi.com/article/10.3390/nu18071159/s1. S1. Group assignment and number of mice per group for in vivo study. S2. Original blots for Western blots. S3. The minimal dataset and the full matrix of p- and F-values for all pairwise comparisons.
