# Supplementary material for: Lilii bulbus Exerts Anti-Seizure Effects by Modulating GABAergic Synapse Organization in the Pentylenetetrazol Kindling Model
Source: Nutrients. 2026 Apr 4;18(7):1159. doi: 10.3390/nu18071159 (PMC13075054; doi:10.3390/nu18071159)
Supplement: Supplementary file 1 [file nutrients-18-01159-s001.zip › nutrients-4172196-supplementary.pdf]

Supplementary Materials

Lilii bulbus Exerts Anti-Seizure Effects by Modulating GABAergic Synapse Organization in the Pentylenetetrazol Kindling Model

Hee Ra Park

Department of KM Science Research, Korea Institute of Oriental Medicine (KIOM), Daejeon 34054, Republic of Korea; hrpark0109@kiom.re.kr; Tel.: +82-42-868-9519; Fax: +82-42-868-9668

S1. Group assignment and number of mice per group for *in vivo* study

Group	Mice	WB/qPCR	Immunostaining	Golgi staining
CON	15 mice	5 mice	5 mice	5 mice
PTZ	15 mice	5 mice	5 mice	5 mice
PTZ+WELB	15 mice	5 mice	5 mice	5 mice
Total 45 mice				

Group	Thy1-GCaMP6s tg mice for Calcium imaging
CON	3 mice
PTZ	3 mice
PTZ+WELB	3 mice
Total 9 mice	

S2. Original blots for Western blots

Figure 3C

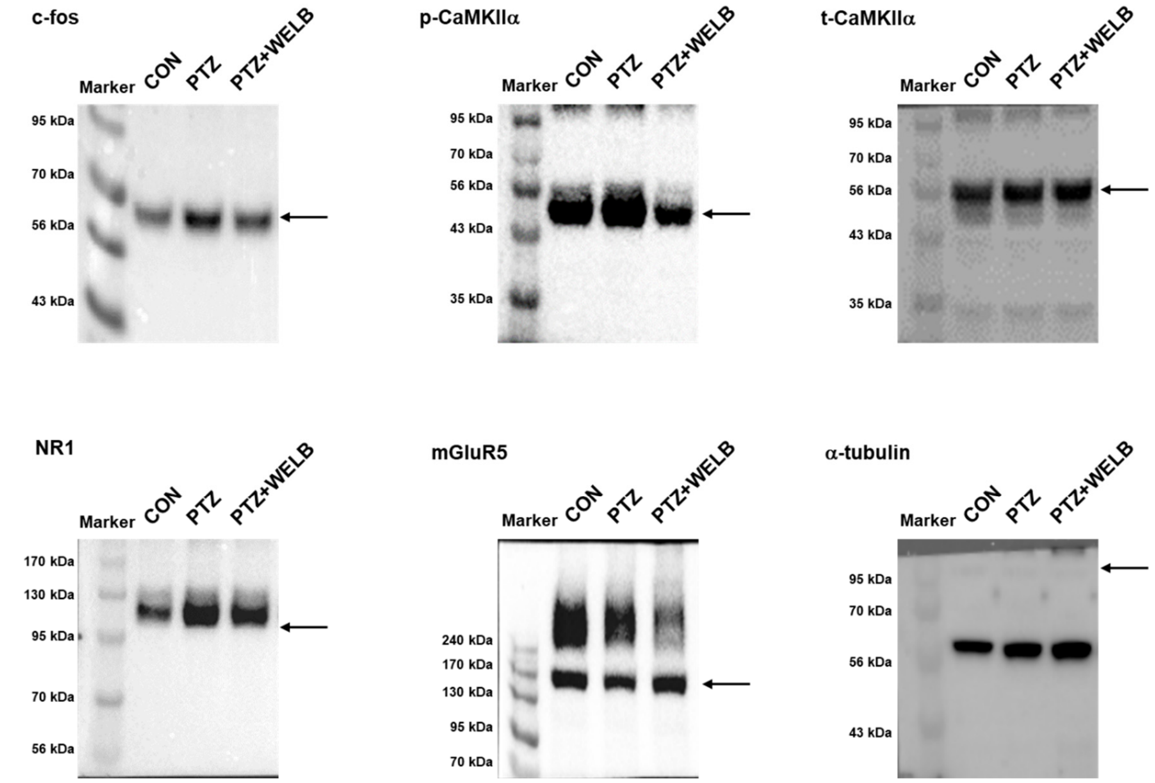

Figure 5A

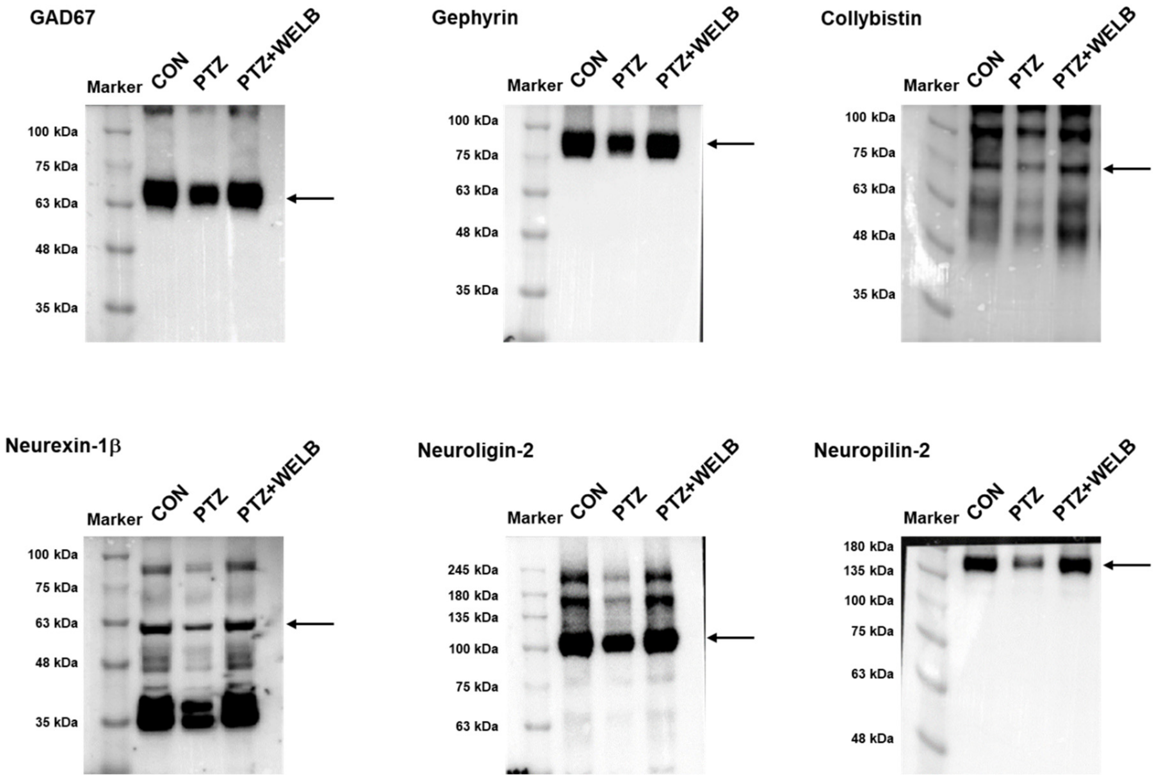

Figure 5A

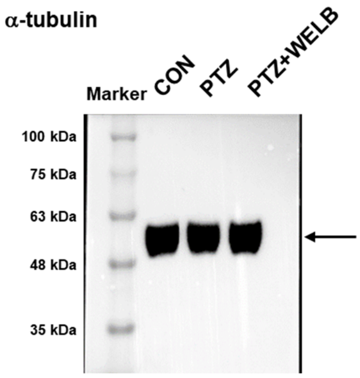
α-tubulin
Marker
CON
PTZ
PTZ+WELB
100 kDa
75 kDa
63 kDa
48 kDa
35 kDa

S3. The minimal dataset and the full matrix of p- and F-values for all pairwise comparisons.

Figure 2A

Two-way ANOVA	Group	CON	PTZ	PTZ+WELB
Kindling stimulation	Interaction<br>F value (DFn, DFd)	P < 0.0001<br>F (24, 546) = 120.1		
	PTZ injection<br>F value (DFn, DFd)	P < 0.0001<br>F (12, 546) = 441.8		
	Group<br>F value (DFn, DFd)	P < 0.0001<br>F (2, 546) = 5077		
1	Mean	0.00	0.47	0.00
	SEM	0.00	0.13	0.00
	Adjusted P value	0.0051		0.0051
2	Mean	0.00	1.03	0.40
	SEM	0.00	0.11	0.13
	Adjusted P value	<0.0001		<0.0001
3	Mean	0.00	1.33	1.07
	SEM	0.00	0.13	0.07
	Adjusted P value	<0.0001		0.1502
4	Mean	0.00	2.13	1.60
	SEM	0.00	0.13	0.13
	Adjusted P value	<0.0001		0.0012
5	Mean	0.00	3.20	2.00
	SEM	0.00	0.11	0.00
	Adjusted P value	<0.0001		<0.0001
6	Mean	0.00	4.13	3.03
	SEM	0.00	0.29	0.13
	Adjusted P value	<0.0001		<0.0001

7	Mean	0.00	5.27	3.20
	SEM	0.00	0.15	0.13
	Adjusted P value	<0.0001		<0.0001
8	Mean	0.00	6.00	3.80
	SEM	0.00	0.00	0.11
	Adjusted P value	<0.0001		<0.0001
9	Mean	0.00	6.00	4.00
	SEM	0.00	0.00	0.17
	Adjusted P value	<0.0001		<0.0001
10	Mean	0.00	6.10	4.33
	SEM	0.00	0.00	0.13
	Adjusted P value	<0.0001		<0.0001
11	Mean	0.00	6.34	4.57
	SEM	0.00	0.13	0.22
	Adjusted P value	<0.0001		<0.0001
12	Mean	0.00	6.40	4.70
	SEM	0.00	0.11	0.22
	Adjusted P value	<0.0001		<0.0001
13	Mean	0.00	6.50	4.77
	SEM	0.00	0.11	0.14
	Adjusted P value	<0.0001		<0.0001

Figure 2B

t-test	Group	PTZ	PTZ+WELB
Latency to clonic seizure	Mean	276.07	637.13
	SEM	23.60	34.43

	Adjusted P value	< 0.0001
	F value (DFn, DFd)	2.128, 14, 14
	t, df	t=8.651, df=28

Figure 2C

t-test	Group	PTZ	PTZ+WELB
Duration of seizure	Mean	32.60	23.87
	SEM	1.76	0.95
	Adjusted P value	0.0002	
	F value (DFn, DFd)	3.434, 14, 14	
	t, df	t=4.363, df=28	

Figure 3B

	Group	CON	PTZ	PTZ+WELB
Calcium imaging	Mean	0.9364	1.851	1.248
	SEM	0.05465	0.1424	0.1034
	Adjusted P value	0.0016		0.0126
	F value (DFn, DFd)	F (2, 6) = 19.10		

Figure 3D

	Group	CON	PTZ	PTZ+WELB
Golgi staining	Mean	14.70	25.80	18.47
	SEM	0.8985	0.9854	0.8076
	Adjusted P value	< 0.0001		< 0.0001
	F value (DFn, DFd)	F (2, 87) = 39.33		

Figure 3F

WB	Group	CON	PTZ	PTZ+WELB
c-fos	Mean	1.042	2.014	0.7400
	SEM	0.03693	0.1234	0.1192
	Adjusted P value	<0.0001		<0.0001
	F value (DFn, DFd)	F (2, 12) = 60.72		
p-CaMKIIα	Mean	1.074	1.580	1.226
	SEM	0.1051	0.07396	0.07827
	Adjusted P value	0.0027		0.0252
	F value (DFn, DFd)	F (2, 12) = 8.928		
NR1	Mean	0.9440	1.600	1.304
	SEM	0.05501	0.09664	0.03311
	Adjusted P value	<0.0001		0.0162
	F value (DFn, DFd)	F (2, 12) = 24.05		
mGluR5	Mean	1.264	1.438	1.272
	SEM	0.08892	0.09260	0.07519
	Adjusted P value	0.2940		0.3232
	F value (DFn, DFd)	F (2, 12) = 1.308		

Figure 4B

	Group	CON	PTZ	PTZ+WELB
GAD67	Mean	16.10	4.500	16.60
	SEM	3.322	1.294	1.684
	Adjusted P value	0.0068		0.0051
	F value (DFn, DFd)	F (2, 12) = 9.045		
VGAT	Mean	1.048	0.5020	1.060

	SEM	0.04104	0.06192	0.0965
	Adjusted P value	0.0003		0.0002
	F value (DFn, DFd)	F (2, 12) = 20.56		
PV	Mean	12.10	3.800	10.90
	SEM	1.409	0.5148	0.6403
	Adjusted P value	<0.0001		0.0004
	F value (DFn, DFd)	F (2, 12) = 22.70		
SOM	Mean	24.00	8.600	19.50
	SEM	3.616	0.3674	2.219
	Adjusted P value	0.0016		0.0159
	F value (DFn, DFd)	F (2, 12) = 10.37		

Figure 4C

	Group	CON	PTZ	PTZ+WELB
Gabra1	Mean	1.194	0.3682	2.348
	SEM	0.05669	0.1165	0.3482
	Adjusted P value	0.0338		<0.0001
	F value (DFn, DFd)	F (2, 12) = 21.50		
Gabra2	Mean	1.391	0.7696	1.867
	SEM	0.1484	0.04420	0.2325
	Adjusted P value	0.0335		0.0008
	F value (DFn, DFd)	F (2, 12) = 11.64		
Gat1	Mean	2.237	0.4617	1.354
	SEM	0.3904	0.08344	0.1108
	Adjusted P value	0.0004		0.0392
	F value (DFn, DFd)	F (2, 12) = 13.76		

Gat3	Mean	1.924	0.5389	1.554
	SEM	0.4540	0.04524	0.1472
	Adjusted P value	0.0076		0.0425
	F value (DFn, DFd)	F (2, 12) = 6.712		
PV	Mean	1.251	0.7199	1.184
	SEM	0.06891	0.08561	0.08822
	Adjusted P value	0.0012		0.0031
	F value (DFn, DFd)	F (2, 12) = 12.62		
SOM	Mean	1.331	0.8666	1.311
	SEM	0.1185	0.06848	0.1538
	Adjusted P value	0.0314		0.0387
	F value (DFn, DFd)	F (2, 12) = 4.877		
CCK	Mean	1.172	0.6678	1.112
	SEM	0.09518	0.06848	0.1538
	Adjusted P value	0.0143		0.0284
	F value (DFn, DFd)	F (2, 12) = 6.085		

Figure 5B

	Group	CON	PTZ	PTZ+WELB
GAD67	Mean	1.132	0.5838	1.064
	SEM	0.03465	0.02709	0.04000
	Adjusted P value	<0.0001		<0.0001
	F value (DFn, DFd)	F (2, 12) = 75.66		
Gephyrin	Mean	1.251	0.6266	1.191
	SEM	0.06586	0.009416	0.03993
	Adjusted P value	<0.0001		<0.0001

	F value (DFn, DFd)	F (2, 12) = 59.17		
Collybistin	Mean	1.203	0.6702	1.048
	SEM	0.06705	0.04900	0.06392
	Adjusted P value	<0.0001		0.0016
	F value (DFn, DFd)	F (2, 12) = 20.53		
Neurexin-1β	Mean	1.257	0.4778	1.264
	SEM	0.07154	0.01452	0.03863
	Adjusted P value	<0.0001		<0.0001
	F value (DFn, DFd)	F (2, 12) = 89.91		
Neuroligin-2	Mean	1.219	0.6618	1.130
	SEM	0.06728	0.03816	0.04679
	Adjusted P value	<0.0001		<0.0001
	F value (DFn, DFd)	F (2, 12) = 32.93		
Neuropilin-2	Mean	1.158	0.5500	1.113
	SEM	0.04852	0.02552	0.04984
	Adjusted P value	<0.0001		<0.0001
	F value (DFn, DFd)	F (2, 12) = 62.66		

Figure 5C

	Group	CON	PTZ	PTZ+WELB
Gephyrin	Mean	1.138	0.5215	1.032
	SEM	0.06462	0.06445	0.1156
	Adjusted P value	0.0005		0.0021
	F value (DFn, DFd)	F (2, 12) = 15.04		
Neurexin-1	Mean	1.175	0.6073	1.194
	SEM	0.09462	0.1107	0.1977

	Adjusted P value	0.0276		0.0232
	F value (DFn, DFd)	F (2, 12) = 5.531		
Neuroligin-2	Mean	1.362	0.6043	1.039
	SEM	0.09496	0.1139	0.1523
	Adjusted P value	0.0017		0.0497
	F value (DFn, DFd)	F (2, 12) = 9.597		
